# Characterisation of the pathogenic effects of the *in vivo* expression of an ALS-linked mutation in D-amino acid oxidase: Phenotype and loss of spinal cord motor neurons

**DOI:** 10.1371/journal.pone.0188912

**Published:** 2017-12-01

**Authors:** Nazanin Rahmani Kondori, Praveen Paul, Jacqueline P. Robbins, Ke Liu, John C. W. Hildyard, Dominic J. Wells, Jacqueline S. de Belleroche

**Affiliations:** 1 Neurogenetics Group, Division of Brain Sciences, Faculty of Medicine, Imperial College London, Hammersmith Hospital Campus, London, United Kingdom; 2 Neuromuscular Diseases Group, Department of Comparative Biomedical Sciences, Royal Veterinary College, London, United Kingdom; University of Edinburgh, UNITED KINGDOM

## Abstract

Amyotrophic lateral sclerosis (ALS) is the most common adult-onset neuromuscular disorder characterised by selective loss of motor neurons leading to fatal paralysis. Current therapeutic approaches are limited in their effectiveness. Substantial advances in understanding ALS disease mechanisms has come from the identification of pathogenic mutations in dominantly inherited familial ALS (FALS). We previously reported a coding mutation in D-amino acid oxidase (DAO^R199W^) associated with FALS. DAO metabolises D-serine, an essential co-agonist at the N-Methyl-D-aspartic acid glutamate receptor subtype (NMDAR). Using primary motor neuron cultures or motor neuron cell lines we demonstrated that expression of DAO^R199W^, promoted the formation of ubiquitinated protein aggregates, activated autophagy and increased apoptosis. The aim of this study was to characterise the effects of DAO^R199W^ *in vivo*, using transgenic mice overexpressing DAO^R199W^. Marked abnormal motor features, e.g. kyphosis, were evident in mice expressing DAO^R199W^, which were associated with a significant loss (19%) of lumbar spinal cord motor neurons, analysed at 14 months. When separated by gender, this effect was greater in females (26%; p< 0.0132). In addition, we crossed the DAO^R199W^ transgenic mouse line with the SOD1^G93A^ mouse model of ALS to determine whether the effects of SOD1^G93A^ were potentiated in the double transgenic line (DAO^R199W^/SOD1^G93A^). Although overall survival was not affected, onset of neurological signs was significantly earlier in female double transgenic animals than their female SOD1^G93A^ littermates (125 days vs 131 days, P = 0.0239). In summary, some significant *in vivo* effects of DAO^R199W^ on motor neuron function (i.e. kyphosis and loss of motor neurons) were detected which were most marked in females and could contribute to the earlier onset of neurological signs in double transgenic females compared to SOD1^G93A^ littermates, highlighting the importance of recognizing gender effects present in animal models of ALS.

## Introduction

Amyotrophic lateral Sclerosis (ALS) is the most common adult-onset neuromuscular disorder, caused by the selective degeneration of motor neurons in the spinal cord, brain stem and motor cortex. The disease usually progresses rapidly leading to severe motor disability with most patients dying within 3–5 years of diagnosis. Understanding the pathogenic mechanisms that underlie the disease has been greatly accelerated by the identification of mutations that cause the familial form of the disease (FALS) [1]. To date at least twenty genes have been found that harbour mutations associated with FALS. The most prevalent mutations are found in *C9ORF72*, *SOD1*, *TAR DNA binding protein 43 (TARDBP)*, and *Fused in Sarcoma (FUS)* and much evidence is accumulating to link these mutations with the classical neuropathological features of both familial and sporadic forms of the disease i.e. the accumulation of cytosolic ubiquitinated protein inclusions, most of which are positive for TDP-43 encoded by *TARDBP* [2]. Interestingly, it has emerged from these genetic studies that dysfunction of two major pathways are strongly associated with ALS pathogenesis. These effects are firstly focussed on components of RNA metabolism, through dysfunction of the regulatory properties of RNA binding proteins affecting splicing and their trafficking between the nucleus and cytosol and secondly proteostasis, affecting the endoplasmic reticulum stress response and protein degradation pathways mediated through the proteasome and autophagy [3]. A less well established area is the understanding of trigger factors that initiate this process which potentially could include glutamate excitotoxicity, impaired calcium homeostasis and oxidative stress.

Recently, our group identified a mutation in the *D-amino acid oxidase* gene (*DAO)* that was transmitted with disease [4], a substitution of arginine by tryptophan at codon 199 (DAO^R199W^), which has shed some light on more upstream events that may trigger disease initiation [4–6]. The selective distribution of the protein in spinal cord and brain stem nuclei suggests a previously unrecognised role for DAO in motor pathways and function [4]. The major function known for DAO is the regulation of the levels of D-serine, which has a fundamental role in excitatory pathways as a co-agonist at the N-Methyl-D-aspartic acid (NMDA) glutamate receptor (NMDAR). This important function of D-serine has now been well characterised and is known to be essential in mediating some actions of glutamate in synaptic plasticity e.g. long term potentiation [7–10]. Furthermore, we have shown that expression of DAO containing the mutation associated with FALS, DAO^R199W^, in primary motor neuron cultures or motor neuron cell lines promotes the formation of ubiquitinated protein aggregates, activates autophagy and increases apoptosis. Importantly, we have shown that these effects can be significantly attenuated by a selective antagonist at the D-serine binding site of the NMDA receptor, using a co-culture system where glial cells expressing DAO^R199W^ are incubated in an insert placed above motor neuron cells lacking the mutation, which recapitulates the effects of the mutation on autophagy and apoptosis in the co-cultured motor neuron cells [6]. In addition, DAO^R199W^ leads to impaired enzymatic DAO enzyme activity in ALS cases carrying the mutation and also when expressed in cell lines [4].

Interestingly, studies of a mouse strain (ddY/DAO^−^)[11] lacking DAO enzyme activity, due to a naturally occurring point mutation at position 181 leading to the substitution of glycine by arginine, revealed that while these animals grow and reproduce normally they exhibit abnormal NMDA receptor-mediated behavour [12]. Furthermore a homozygous mouse line expressing this mutation was generated (DAO^-^/^-^) that developed an abnormal limb reflex and a significant loss of motor neurons in lumbar spinal cord at 8 months [13]. The study also showed that both in this transgenic line and in the SOD^G93A^ mouse model of ALS, D-serine accumulated in the spinal cord during disease progression and a marked suppression in DAO activity was seen in the reticulospinal tract, a pathway that plays an important role in regulating motor neuron excitability [13].

The aim of this study was to determine whether the deleterious effects of DAO^R199W^ on motor neurons detected in cell culture could be seen *in vivo* in transgenic mice overexpressing DAO^R199W^. In addition, we crossed the DAO^R199W^ transgenic mouse line with the SOD1 mouse model of ALS to generate double transgenic mice expressing both mutations (DAO^R199W^/SOD^G93A^) to determine whether their effects were synergistic. We found that significant motor neuron loss occurred in DAO^R199W^ transgenic mice but the presence of DAO^R199W^ in double transgenic mice (DAO^R199W^/SOD^G93A^) did not potentiate the effects of SOD1 over-expression on survival.

## Results

### Generation of a new line of transgenic animals overexpressing DAO^R199W^

**Fig 1:**
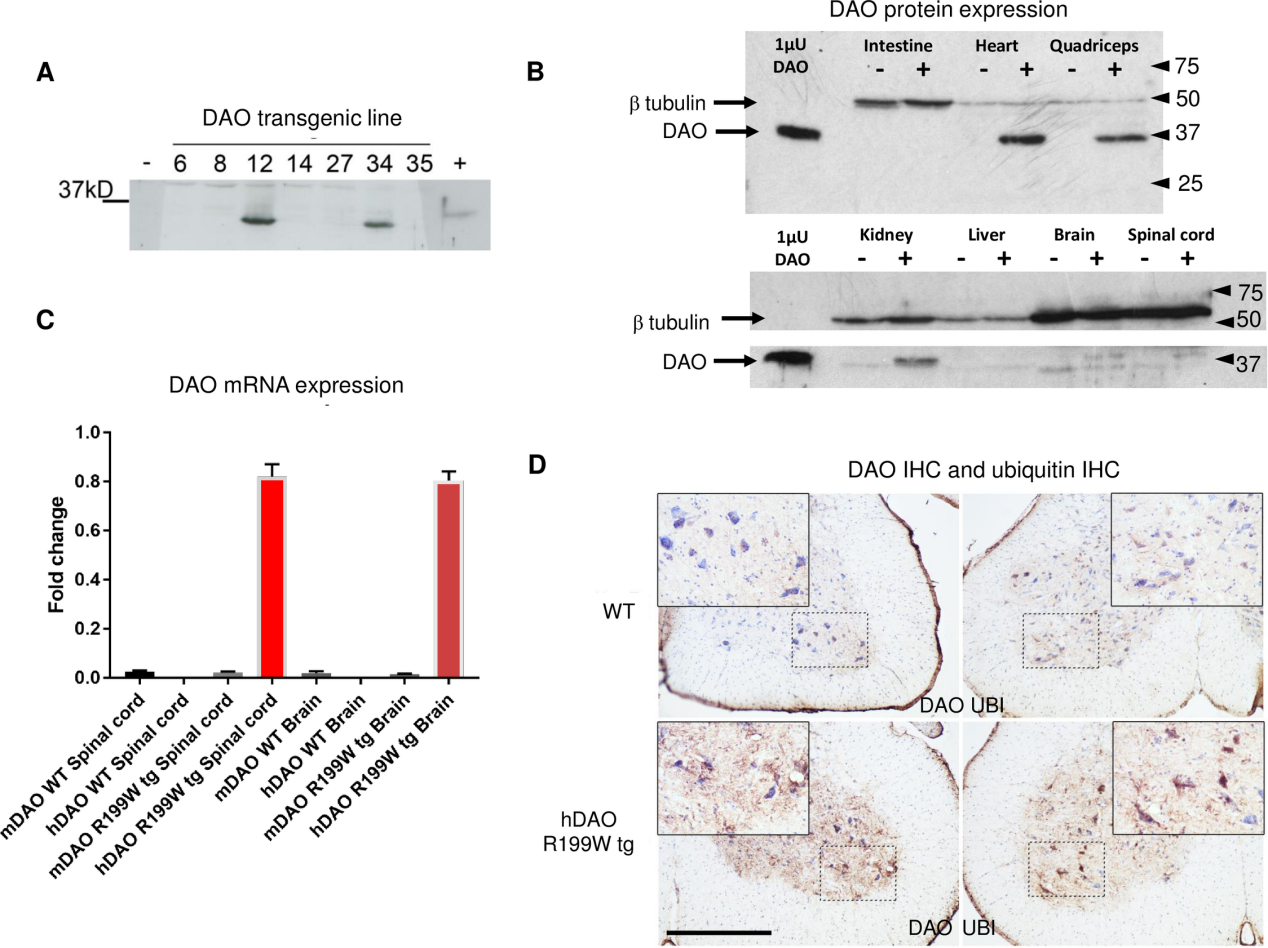
**Transgene expression in two mouse lines (A), regional tissue expression of DAO protein (B) and mRNA (C) in DAO**^**R199W**^ **transgenic mice and immunohistochemical localisation of DAO and ubiquitin in DAO**^**R199W**^ **Transgenic mice (D). A. Expression of DAO in spinal cord from two DAO**^**R199W**^ **lines.** Western blot analysis of different transgenic mouse lines expressing human DAO. 200ng of spinal cord from each animal was separated by SDS-PAGE and blotted with anti-DAO antibody. Expression of DAO was present in two DAO^R199W^ transgenic mice lines, 12 and 34 (used in the survival analysis). A porcine DAO protein standard (indicated by a + sign) was loaded in the final well. B. **Regional expression of DAO protein in DAO**^**R199W**^ **transgenic mice**. The highest concentrations of DAO (37kDa) in DAO^R199W^ transgenic mice (indicated by +) were found in heart and quadriceps muscle (upper panel), followed by kidney, spinal cord, brain (lower panel) and in both lines (shown here for line 34) compared to wild-type mice (indicated by a -). Lower levels were found in liver and small intestine. The loading control was beta tubulin (50kDa). DAO immunoreactivity was low in non-transgenic mice. C. **Regional expression of DAO mRNA in DAO**^**R199W**^ **transgenic mice**. Expression of human DAO (hDAO) and mouse DAO (mDAO) in spinal cord, brain and quadriceps muscle from wild-type (WT) and DAO^R199W^ transgenic mice (R199W tg). D. **Spinal cord expression of DAO and ubiquitin in DAO**^**R199W**^ **Transgenic mice.** Lumbar spinal cord sections (L2-L4) from 8 month old non-transgenic and R199W DAO tg mice were immunostained for DAO and ubiquitin and counterstained with Nissl (blue). Ventral horns with their enlargement (boxed area) are shown for each section. Spinal cord sections from the DAO^R199W^ transgenic showed widespread distribution of DAO staining (brown) throughout the ventral grey matter (lower left hand panel) particularly in dendrites and interneurons with lower levels present in non-transgenic littermates (upper left hand panel). Similarly, ubiquitin staining was more intense in sections from DAO^R199W^ transgenic mice (lower right hand panel) compared to their non-transgenic littermates (upper right hand panel). Scale bar, 500um.

Two independent lines were generated and upon successful transmission, transgene expression was confirmed in spinal cord using western blot analysis (Fig 1A) and extended to a wider range of tissues (Fig 1B). As DAO^R199W^ protein was under the influence of the CAGG promoter/enhancer construct (beta actin promoter with the CMV enhancer), widespread expression in many tissues was seen. The highest levels of DAO expression were found in muscle and heart, followed by kidney, spinal cord and brain, under conditions where endogenous levels of DAO were low or undetectable. Quantitative PCR using selective primer sets that detect expression of the human DAO transgene in DAO^R199W^ transgenic mice indicated that the DAO^R199W^ transgene was present at concentrations 40-fold greater than that of mouse DAO in the same animals (Fig 1C). In keeping with protein detection by Western blot analysis, the DAO^R199W^ transgene was markedly over-expressed in spinal cord compared to wild-type controls and was present at even greater levels in skeletal muscle and heart. We also investigated DAO activity in DAO^R199W^ transgenic mice compared to control littermates using the peroxidase-linked colorimetric conversion of ortho-dianisidine (protocol modified from Tedeschi *et al*)[14] and found a mild reduction in overall enzyme activity in the brain and spinal cord, suggesting that a degree of hetero-dimerisation with endogenous protein may occur (**Figure A in S1 File**). No enzyme activity was detected in the muscle of wild-type and transgenic mice despite substantial expression at the mRNA level in the transgenic mice.

DAO immunoreactivity was abundant in DAO^R199W^ transgenic mice compared to control littermates (Fig 1D) being present in large motor neurons and the surrounding grey matter of ventral spinal cord. Similarly, ubiquitin immunoreactivity was high in grey matter of the ventral spinal cord in DAO^R199W^ mice, being most abundant in the cell bodies of large motor neurons. In contrast, levels of ubiquitin were only present at low levels in controls (Fig 1D), confirming previous studies in NSC-34 cell lines overexpressing DAO^R199W^ compared to DAO^WT^ where it was established that a prominent feature of the cells expressing the mutant DAO^R199W^ allele was the presence of ubiquitinated protein aggregates [4].

### Characterisation of the DAO^R199W^ transgenic line: Effect on body weight and morphological phenotype

**Fig 2:**
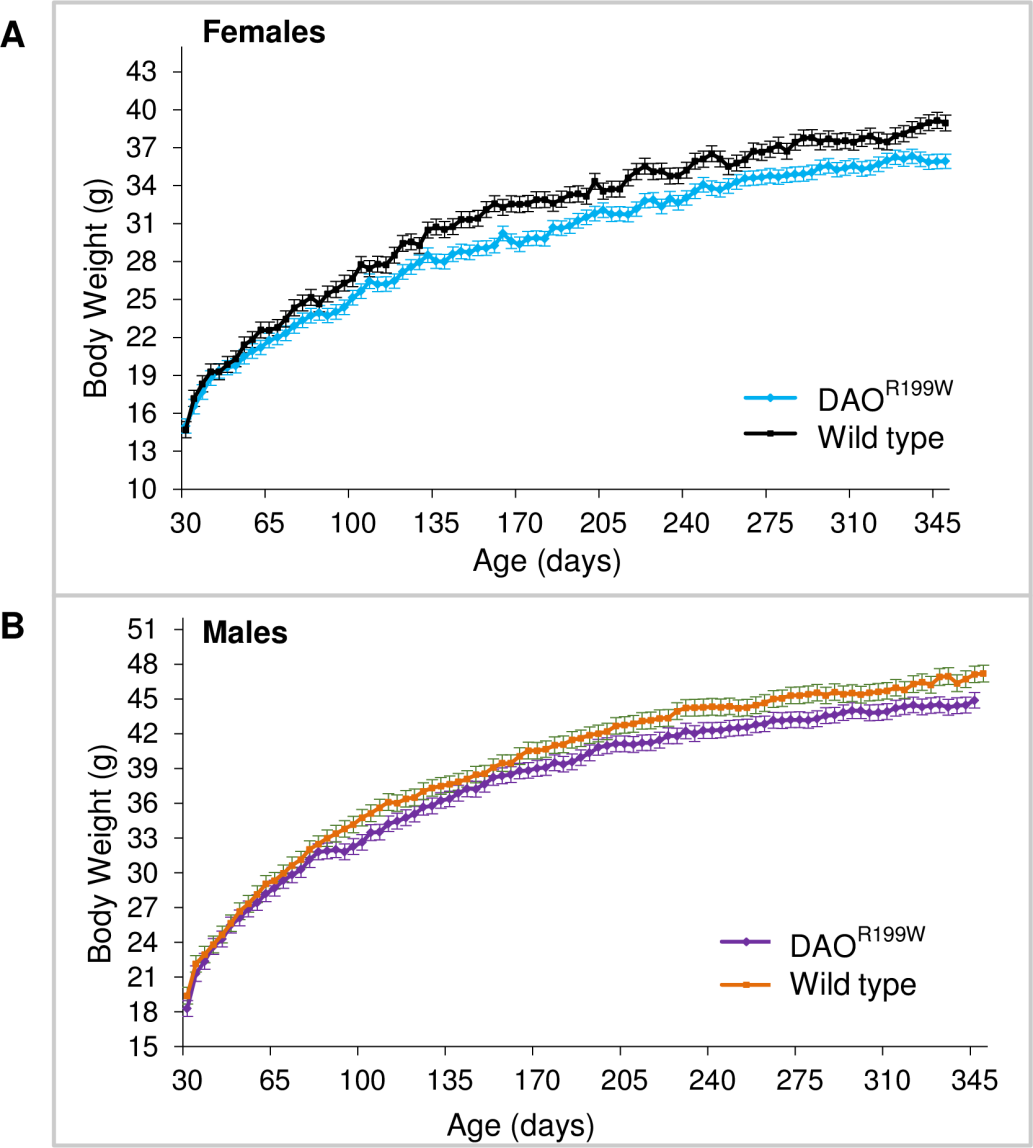
Longitudinal assessment of body weight in DAOR199W and wild-type littermates. Mean group body weight plotted over time from 30 days to 350 days of age. (A) females, n = 23 DAO^R199W^ (blue) and (n = 17) non-transgenic littermates (black). (B) males, n = 15 DAO^R199W^ (purple) and (n = 16) non-transgenic littermates (orange). Values are means± SEMs.

Initially, we carried out a longitudinal large cohort survival study over a period of one year, during which time body weight, motor features and gait were monitored. The assessor was blinded to the genotype of the animals. Animals expressing the transgene consistently had a lower body weight at each age compared to their wild type littermates, which was evident in both males and females (Fig 2A and 2B).

**Fig 3:**
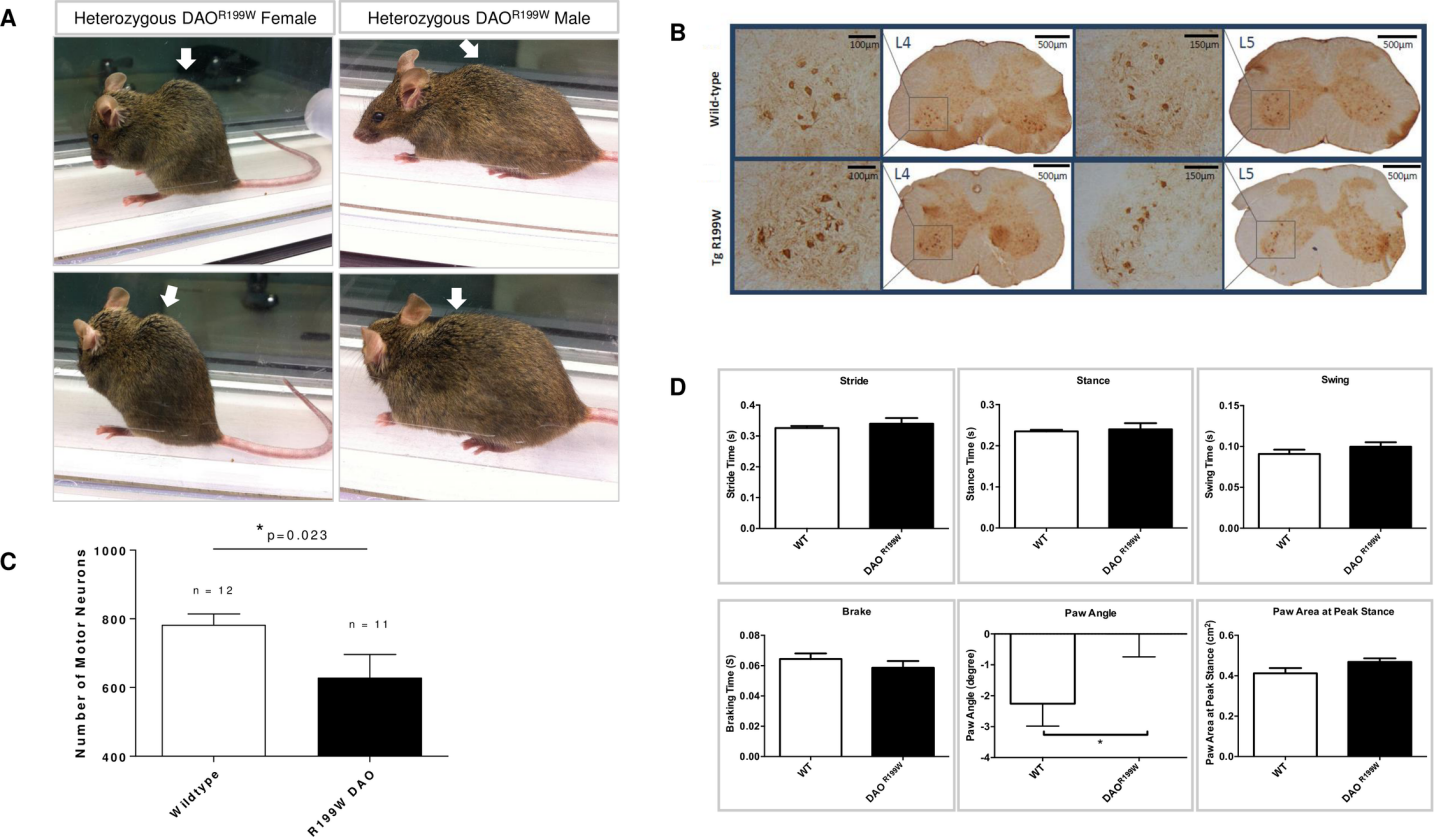
**Phenotype of R199W DAO mouse showing (A) Kyphosis, (B) motor neuron loss in lumbar spinal cord, (C) ChAT immunohistochemisty in lumbar spinal cord (D) Assessment of various gait indices in the hindlimbs of female DAO^R199W^ and wild-type littermates.** (A) Kyphosis of the spine demonstrated in both male and female DAO^R199W^ transgenic lines. The arrow denotes kyphosis of the spine. (B) Motor neuron counts in wildtype and R199W DAO mice aged 14–15 months. Lumbar motor neurons in regions L3-L5 were stained with anti-ChAT antibody and every third section quantified for both male and female wildtype and R199W DAO mice. The number of motor neurons in R199W DAO mice was reduced by 19.7% compared to wild-type mice (one-tailed, unpaired t-test, *p<0.05). When split by gender, motor neuron loss was significantly reduced by 25.7% in females (p = 0.0132*, one-tailed, unpaired t-test) but in males the reduction was by 16.5%, which was not significant. Histograms show means for 40 sections per animal ± SEM. (C) ChAT immunohistochemistry of L4 and L5 regions of a wild-type and transgenic animal with a DAO^R199W^ mutation. Immunohistochemistry was performed on the lumbar spinal cord using anti-ChAT on 20μm sections of spinal cord. (A) Wild-type female L4 and L5 sections. (B) Female with DAO R199W mutation L4 and L5 sections. The number of ChAT-stained motor neurons in the female mice was 25.7% lower for the transgenic group compared to the wild-type group. (D) Assessment of various gait indices in the hind-limbs of female DAO^R199W^ and wild-type littermates. Mean groups combined, plotted for hind-limbs of the DAO^R199W^ (black bars) and WT (white bars) female mice. This was done through averaging the individual values generated by the DigiGait system for each hind-limb of a particular animal (*n* ≥ 10 per experimental group). Stride (sum of stance and swing), stance (weight bearing portion of the stride in which the paw remains in contact with the belt), swing (when the paw is not in contact with the belt) presented in the top panel, with P values of 0.5238, 0.7560 and 0.273 respectively (two-tailed, unpaired t-test, P < 0.05). Brake (time intervals between initial and maximal paw contacts with the belt), paw angle (angle of the hind-paw during peak stance in relation to the long axis of the body) and paw area at peak stance (maximal paw area in contact with the treadmill during the stance phase) presented in the bottom panel, with P values of 0.4509, 0.0435*, 0.0673 respectively (Mann-Whitney test carried out for brake time and two-tailed, unpaired t-test for the other two parameters, P < 0.05). Asterisk indicates a significant difference in term of the degree of paw angle between WT controls and DAO^R199W^ mice. Values are standard error of the mean (±SEM).

We observed some substantial and distinct features present in DAO^R199W^ animals in terms of physical appearance that were absent from wild-type littermates (Fig 3A). **All** males and females bearing the mutant transgene appeared very hunched and developed progressive kyphosis, which was more conspicuous in transgenic females than the males.

### Characterisation of the DAO^R199W^ transgenic line: Effect on motor neuron populations

The effect of the DAO^R199W^ mutation on motor neuron cell survival was investigated in the DAO^R199W^ transgenic line by quantification of the motor neurons in the lumbar region of the spinal cord at the age of 14 months (13.5 to 14.5 months, Fig 3B and 3C). The quantification of the ventral horn motor neurons (L3-L5) of the transgenic animals versus their wild type littermates (*n* ≥ 5 per genotype, per sex) revealed a significant reduction in motor neuron numbers in mice carrying the mutant allele in comparison to their wild type littermates. When separated according to gender, this decrease (25.7%) was significant in females (P = 0.0132) though not in males (16.5% reduction).

### Characterisation of the DAO^R199W^ transgenic line: Gait analysis

The phenotypic difference between the wild type and mutant animals led us to design a gait analysis experiment. In this study, we analysed both genotypes for their gait, footfall, and mechanics covering over 40 parameters but observed no significant differences in gait and body movement between DAO^R199W^ animals and their wild type littermates. A significant difference was however observed in terms of hind-paw angle in the DAO^R199W^ transgenic females (P = 0.0435*, *n* ≥ 10) (Fig 3D). Paw angle is defined as the angle of the paw (hind or fore) during peak stance in relation to the long axis of the body. Hence, a negative value for this parameter indicates that the animal places its paw inward towards the long axis of its body every time the paw is placed on the surface of the treadmill, and a positive value is an indication of the paws being placed outwards away from the long axis of the body [15–17].

The DAO^R199W^ females place their hind-paws in a straight line, almost parallel to the long axis of their bodies (mean value of angle, -0.008333 ± 0.7330°) in comparison to their wild type littermates (mean value of angle,-2.255 ± 0.7275°). The data obtained for the same parameter for the forepaws suggested that in order to maintain balance due the changes in the paw angle, the animal was forced to turn its forepaws inwards, giving a mean value of -1.241 ± 0.5678°, compared to -0.005000 ± 0.5485° in wild type littermates (forepaws almost parallel to the body axis) (Fig 3D). These differences may reflect an adaptation to weight loss, muscle atrophy and skeletal changes.

In summary, while no overt ALS phenotype was evident in mice expressing DAO^R199W^, marked structural and abnormal motor features were evident which were associated with a significant loss of lumbar motor neurons. While a mutation can cause a lethal disease in humans, it may not be possible to trigger the same phenotype in a transgenic mouse models carrying the same mutation, as seen for VAPB mutations [18], and hence we investigated whether the DAO^R199W^ mutation would potentiate the effects of the SOD-1 mouse model of ALS.

### DAO^R199W^ and SOD1^G93A^ survival study

Given the well-known nature of the SOD1^G93A^ mouse model and its well-defined lifespan, we examined the effect of the DAO^R199W^ mutation on survival of SOD1^G93A^ mice in a longitudinal large cohort survival study to see if the mutant DAO increased the rate of disease development and progression. Thus we crossed heterozygous DAO^R199W^ females with SOD1^G93A^ male transgenic mice. Animals with SOD1^G93A^ and DAO^R199W^/SOD1^G93A^ genotypes were recruited in the survival study, where the operator was blind to the genotype of each mouse. The development of ALS related disabilities in both genotypes was assessed twice a week using a neurological scoring system (with a scale of 0 to 4, where 0 was normal and four represented complete paralysis of both hind-limbs) commencing from 30 days of age in conjunction with the assessment of body weight [19]. The end point was established as the time at which the mouse was unable to right itself within 20 seconds when placed on its side and at this stage the animal was culled and the final age and weight recorded [19, 20].

**Fig 4:**
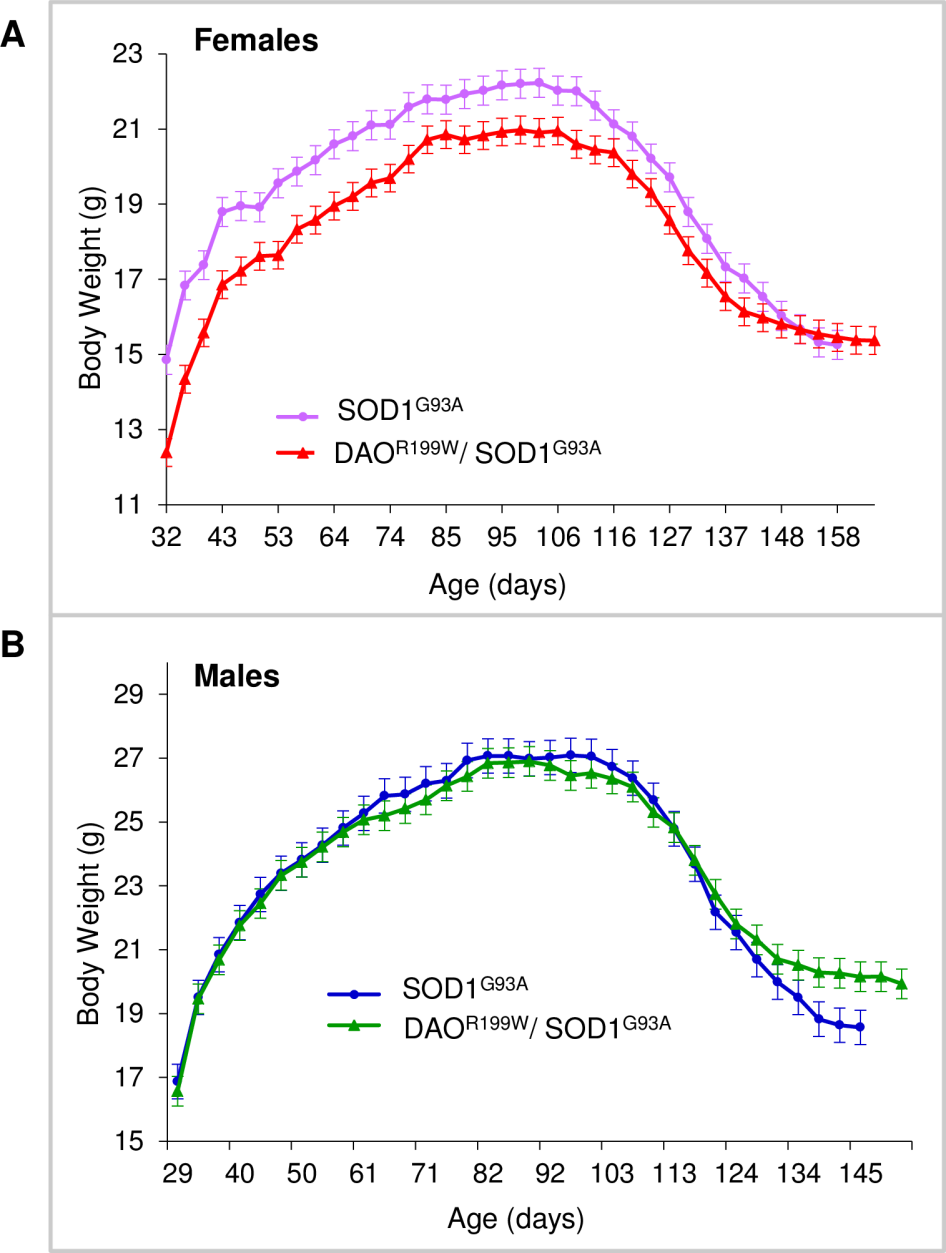
Longitudinal assessment of body weight in female SOD1^G93A^ and double transgenic (SOD1^G93A^/DAO^R199W^) littermates. Mean group body weight plotted for SOD1^G93A^ and double transgenic (SOD1^G93A^/DAO^R199W^) mice over time from approximately 30 days of age until all the animals within a group reached the end stage and the study was terminated (*n* ≥ 14 per experimental group). As mice within groups died at different ages, in order to avoid decomposition of the mean at later stages, the final weights of all mice were carried forward when calculating group mean values, until the date at which the last mouse within each group died. Values are means with the standard error of the mean (SEM) indicated by error bars. (A) SOD1^G93A^ females are presented in purple, and the double transgenic (SOD1^G93A^/DAO^R199W^) littermates are displayed in red. (B) SOD1^G93A^ males are presented in blue, and the double transgenic (SOD1^G93A^/DAO^R199W^) littermates are displayed in green.

Assessment of body weight demonstrated the expected gender differences, male animals being heavier in terms of total body weight compared to their female littermates. Both genotypes, SOD1^G93A^ and DAO^R199W^/SOD1^G93A^, increased weight steadily until all animals reached the maximum peak weight at approximately 88 days for males and 99 days for females (Fig 4A and 4B). After this point all animals experienced a marked decrease in their body weight, which continued to the humane end point. The most notable difference between genotypes was the decreased body weight seen in double transgenic female animals compared to SOD1 mice at all stages up until the peak weight was attained.

### Assessment of neurological scores

**Fig 5:**
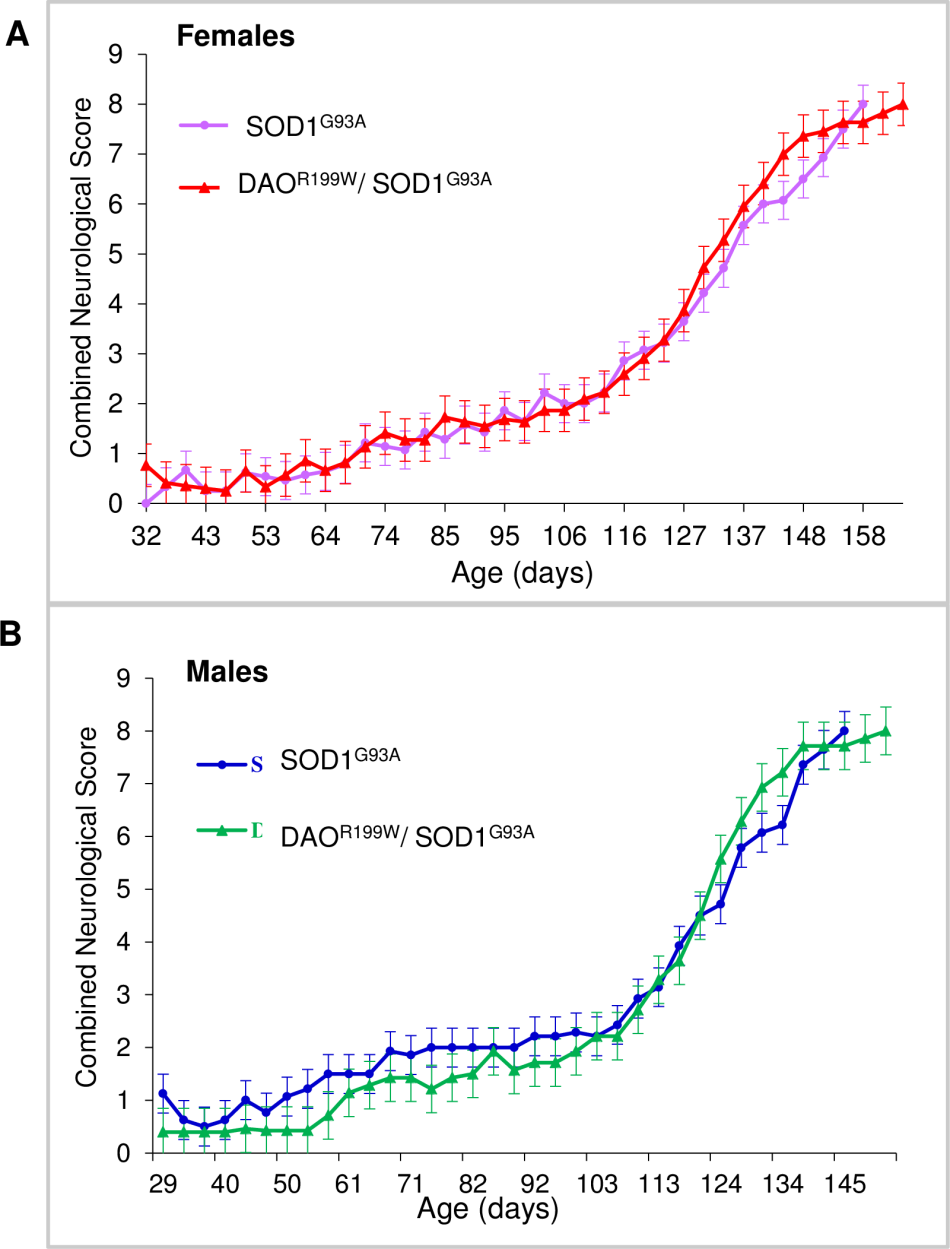
**Longitudinal assessment of neurological scores in female SOD1^G93A^ and double transgenic (SOD1^G93A^/DAO^R199W^) littermates (A) and for male mice (B).** Mean group combined neurological score plotted for SOD1^G93A^ and double transgenic (SOD1^G93A^/DAO^R199W^) mice over time from approximately 30 days of age until all the animals within a group died and the study was terminated (*n* ≥ 14 per experimental group). A combined neurological score was calculated for each mouse at each time point through summation of the neurological scores recorded for its left and right hind-limbs. Values are means with standard error of the mean (SEM) indicated by error bars. (A) SOD1^G93A^ females are presented in purple, and the double transgenic (SOD1^G93A^/DAO^R199W^) littermates are displayed in red. (B) SOD1^G93A^ males are presented in blue, and the double transgenic (SOD1^G93A^/DAO^R199W^) littermates are displayed in green.

Analysis of neurological scores for both females and males demonstrates that both genotypes appeared normal early in their lifespan and did not develop any abnormalities until approximately 70 days of age, after which neurological scores consistently worsened until the animals reached the end stage. No significant differences were seen between the two genotypes (Fig 5).

Further analysis of the neurological scores was carried out as previously described [21] to yield values for onset of neurological signs, disease onset, disease progression and survival. Onset of neurological symptoms was defined as the point in time at which the animal reached a score of 2 (displayed by toe curling or dragging any part of the foot). Double transgenic females first displayed a neurological score of 2 at approximately 125 days of age, significantly earlier (p = 0.0239) than their SOD1^G93A^ littermates (~131 days). Double transgenic males reached the score of 2 at approximately 119 days, which is slightly (but not significantly) later than their SOD1^G93A^ littermates (~113 days). In addition, comparison of the same genotypes across the sexes indicate that while there were no differences in terms of the onset of neurological symptoms between double mutant transgenic mice, there was a significant difference in the time of onset between the SOD1^G93A^ animals, with females exhibiting significantly later onset than male littermates (Table 1).

**Table 1:** Kaplan Meier time to event analyses for SOD1^G93A^ and double transgenic (SOD1^G93A^/DAO^R199W^) in both males and females. A. Median values obtained from Kaplan-Meier analysis for the time taken for mice to reach a score of 2 in both hindlimbs (onset of neurological scores), age at maximum weight (disease onset), disease progression and time taken for animals to reach the humane end point determined by its inability to right itself within 20 seconds of being placed on its side (survival) for both males and female littermates of either genotype groups. B. The Log-rank test assesses the null hypothesis that the Kaplan-Meier curves for all groups are identical (i.e. the presence of the DAO^R199W^ transgene did not change the time taken to reach the event being analysed). Thus, low P-values (shown below) indicate that differences between genotype groups did not occur due to chance. Log-rank analysis of the onset of neurological symptoms in females carrying the SOD1G93A transgene versus their double transgenic littermates revealed that there were significant differences between the groups for this particular measurement. A significant difference was also detected in survival between the double transgenic males and females via the log-rank test. Significant differences are indicated in bold. Threshold of significance was set at P < 0.05.

**(A)**	**Median (days)**			
	**SOD1**^**G93A**^		**Double Transgenics (DT)**	
	**Males**	**Females**	**Males**	**Females**
**Onset of Neurological Symptoms**	113	**131**	119	**125**
**Disease Onset**	89	99	88	99
**Disease Progression**	42	41	39	42
**Survival**	129	136	126	135
**(B)**	**Log-Rank Test (*P* Value)**			
	**SOD1**^**G93A**^	**DT**	**DT vs. SOD1**^**G93A**^	
	**Males vs. Females**	**Males vs. Females**	**Males**	**Females**
**Onset of Neurological Symptoms**	**0.0004**	0.0518	0.3865	**0.0239**
**Disease Onset**	0.1102	0.1131	0.8183	0.7358
**Disease Progression**	0.5786	0.3318	0.2935	0.4750
**Survival**	0.0771	**0.0241**	0.2229	0.8242

Analysis of disease onset, defined as the point in time the animal was at its highest weight, showed an earlier age of 89 days in males compared to females (99 days of age). Onset of disease in both SOD1^G93A^ and double transgenic mice was very similar (Table 1). Progression of disease, defined as the period of time between disease onset and the end stage, was very similar across all groups, ranging from 39 to 42 days.

Survival time, defined as the period of time between birth and the humane end point, determined by the inability of the animal to right itself within 20 seconds of being placed on its side, showed no major differences between the different genotypes and gender (Table 1), SOD1^G93A^ and the double transgenic mice lived for a median of 129 and 126 days respectively in males and for 136 and 135 days respectively in females.

Comparison of the survival curves across the genotypes indicated that SOD1^G93A^ females had a longer average lifespan (136 days) than their SOD1^G93A^ male littermates (129 days) (Table 1) but these differences did not reach significance (P = 0.0771). Similar results were obtained for the double transgenic animals, with females living significantly longer (median of 135 days, P = 0.0241) than their double transgenic male littermates (median of 126 days).

## Discussion

In this study we have characterized the *in vivo* effects of expression of a pathogenic mutation in DAO that is associated with familial ALS [4]. In earlier studies in cell culture, we showed that overexpression of DAO^R199W^ promoted apoptosis in both primary motor neuron cultures and the motor neuron cell line, NSC-34, whereas overexpression of the wild-type protein DAO^WT^ was without effect on cell survival. Other changes occurring in motor neurons expressing the mutant protein included the formation of ubiqutinated protein aggregates and substantial activation of autophagy [6]. Although these pathogenic effects may be mediated primarily by the accumulation of the mutant protein, other factors may be relevant, such as the impaired activity of DAO and consequential effects on D-serine metabolism. D-serine is a co-agonist at the NMDA receptor playing an essential role in mediating excitatory activity in the CNS. We showed that co-culturing motor neurons with glial cells expressing DAO^R199W^ was sufficient to induce apoptosis in motor neurons lacking the mutant protein and that this effect was sensitive to 5,7-Dichloro-4-hydroxyquinoline-2-carboxylic acid (DCKA), an NMDAR antagonist selective for the D-serine/glycine site [6]. The increased protein aggregation and upregulation of autophagy seen in motor neuron cell lines expressing DAO^R199W^ compared to cell lines expressing DAO^WT^ were both also sensitive to this antagonist. These results suggest that the pathogenic effects of DAO^R199W^ may at least be partly mediated through an excitotoxic mechanism, potentiated by the build-up of D-serine in the synapse. The high levels of DAO found in human spinal cord and brain stem motor neurons are consistent with the vital role played by DAO in preventing excitotoxic cell death in these regions.

In this study, we have generated transgenic mouse lines expressing the human DAO mutation associated with FALS in order to characterise the effects of this mutation *in vivo*. We monitored body weight, motor features, gait and quantified motor neurones in lumbar spinal cord in transgenic lines expressing DAO^R199W^ in a longitudinal large cohort survival study over a period of one year to characterise effects on motor function. The most distinctive features seen were decreased body weight, marked kyphosis and loss of motor neurons in spinal cord. All of these features are characteristic of several ALS models including SOD1^G93A^ transgenic mice [22] and conditional targeted inactivation of the *Tardbp* gene in spinal cord motor neurons [23]. In addition, recent studies of a naturally occurring mouse mutation in DAO [11] DAO^G181R^ [24], bred as a homozygous line exhibited marked effects on motor phenotype, with abnormal reflexes characterised by retraction of hind limbs, as occurs in SOD1^G93A^ mice, accompanied by a significant reduction in motor neuron number, as occurred in this study in DAO^R199W^ mice, and axonal degeneration with muscle atrophy demonstrated at 15 months [13].

Animals expressing the DAO^R199W^ transgene consistently had a lower body weight at each age compared to their wild type littermates (Fig 2), which was evident in both females and males (Fig 2A and 2B). This clearly shows that impaired DAO activity affects normal weight gain in heterozygous animals expressing the mutant protein in the presence of endogenous mouse DAO. The decreased body weight is consistent with studies on ddY/DAO− mice that lack DAO activity. When fed a diet in which L-phenylalanine was replaced by D-phenylalanine, these mice were unable to maintain their body weight compared to wild type animals (ddY/DAO+) [25]. This study clearly shows that impaired DAO activity affects normal weight gain both when given a D-amino acid enriched diet and also in our study on heterozygous animals expressing the mutant protein without D-amino acid enrichment but in the presence of endogenous mouse DAO. Both males and females bearing the mutant transgene also developed progressive kyphosis, which was more conspicuous in transgenic females than the males. This skeletal abnormality is also prominent in mouse models of ALS such as the SOD1 mouse carrying the human ALS mutation and may be a consequence of the weakening of the extensor thoracic paraspinal muscles as suggested by Wu et al [23] and as seen in ALS patients [26]. Further studies of gait and body movement in DAO^R199W^ animals demonstrated a significant difference in terms of paw angle in the DAO^R199W^ transgenic females in comparison to their wild type littermates which is likely to have been a consequence of degenerative changes resulting from motor neuron loss and muscle atrophy.

Although D-serine was not quantified in tissue homogenates in transgenic animals in this study, D-serine immunoreactivity was measured in lumbar spinal cord in transgenic and wild-type littermates using a rabbit polyclonal anti-serine antibody raised against D-serine crosslinked to bovine serum albumin with glutaraldehyde (Abcam, dilution 1:5,000), as previously used to characterise D-serine distribution in human and mouse spinal cord [6], where the full characterisation is described. Consistent with these studies, we found that D-serine immunoreactivity was predominantly concentrated in large motor neurons in transgenic mice and their non-transgenic littermate (**Figure B in S1 File**). In addition, serine racemase was abundant in spinal cord grey matter and strongly localised to large motor neurones (**Figure C in S1 File**).A similar distribution was seen in both wild-type and DAO^R199W^ mice but this did not reflect changes occurring in extracellular and non-neuronal compartments or in the relative levels of D and L-serine. However, it is already known that D-serine is elevated in spinal cord of sporadic cases of ALS and also in the SOD1 mouse model of ALS suggesting the involvement of glutamate excitotoxicity in ALS pathogenesis [27]. Furthermore the same group also showed that D-serine was significantly elevated in spinal cord tissue homogenates from the DAO^G183R^ homozygous mouse line at 5 months, using a sensitive and selective 2D-HPLC method, but a significant increase was not detected in the heterozygous mouse line [13].

In summary, despite the development of motor defects, within the time frame of this study, overall survival was not reduced in DAO^R199W^ mice. This suggests that other factors must contribute to the severe phenotype seen in ALS cases expressing this mutation. We therefore looked to see if the presence of DAO^R199W^ potentiated the development of disease in SOD1^G93A^ transgenic mice by crossing the DAO^R199W^ and SOD1^G93A^ mouse lines to generate a double transgenic line (DAO^R188W/SOD1G93A^). A role for DAO in the pathogenicity of the SOD1^G93A^ mouse has already been demonstrated by the observation that DAO activity is decreased by 42%, which is equivalent to the reduction seen in DAO heterozygote mice [13]. The effect of this reduced DAO activity, measured using a sensitive 2D-HPLC method, was also confirmed to result in the expected increase in levels of D-serine in spinal cord that increased with disease progression [13].

Body weight and neurological scores were monitored twice a week and survival was determined as the age at which the animal was unable to right itself within 20 seconds of being placed on its side. Overall, both the SOD1^G93A^ and their double transgenic littermates followed the same pattern in terms of their marked reduction in body weight after 90 days. However, marked differences in body weight were seen between males and females (Fig 4). Whilst body weight was lower in female double transgenic mice compared to SOD1^G93A^ mice, in males both SOD1^G93A^ and double transgenic animals showed a similar body mass (Fig 4). This differential effect in females may reflect an endocrine effect, as testosterone is known to stimulate DAO activity in mouse kidney [28].

Since the DAO^R199W^ transgenic line is heterozygous for the DAO allele on the C57B16xCBA/Ca background, as well as expressing the mutant allele they also express the wild type endogenous DAO protein. It has been demonstrated that DAO activity is significantly reduced in the lumbar region of the SOD1^G93A^ mice, to levels approaching those of the ^B6^DAO^+/−^ mice [13]. It is thus likely that the effect of the endogenous DAO protein is suppressed by the presence of the SOD1^G93A^ transgene in our double transgenic animals. As DAO is required for degradation of D-amino acids, it is therefore possible that the presence of both the SOD1^G93A^ and DAO^R199W^ mutant transgenes in the double transgenic females suppressed or diminished the already low levels of endogenous DAO enzyme activity, preventing animals from maintaining their overall body mass and consequently exhibiting a consistent reduction in weight when compared to their SOD1^G93A^ littermates throughout their lifespan (Fig 2). Conversely, the marked similarity in body weight between the double transgenic males and their SOD1^G93A^ littermates could stem from the innately higher levels of DAO enzymatic activity found in males compared to females which might buffer the influence of the double transgene, permitting a residual level of function sufficient for the animals to maintain their body weight at a level comparable to their SOD1^G93A^ littermates. This low level of endogenous DAO enzyme activity could also account for the attenuated motor neuron loss in the lumbar region of the spinal cord that we found in males which (in contrast to the reduction observed in females) did not reach significance. This in turn may be an indication of protective properties of the wild type DAO protein against motor neuron degeneration.

Interestingly, onset of neurological symptoms in double transgenic mice also showed a differential effect in males and females (Fig 5, Table 1). Most notably, while males of both genotypes typically developed symptoms at earlier time points than females, female double transgenic animals displayed a symptomatic onset six days earlier than their female SOD1^G93A^ littermates (125 days vs 131 days, P = 0.0239). No such potentiation was observed in male double transgenic mice. Disease progression and survival appeared largely insensitive to the presence of the DAO^R199W^ transgene, although double transgenic females did survive significantly longer than double transgenic males (P = 0.0241).

In conclusion, our results indicate that although the different genotypes exhibit some clear differences, overall the presence of the DAO^R199W^ transgene alongside SOD1^G93A^ did not exert any major effects on disease onset, progression or survival in either sex. This is likely due to the aggressive and accelerated nature of the phenotype enforced by the SOD1^G93A^ mutation, masking any effect the DAO^R199W^ mutation may trigger. It is also important to point out that in our initial characterization of the DAO^R199W^, we detected enhanced ubiquitination at 8 months (Fig 1C) and motor neuron cell loss at 14 months (13.5 to 14.5 months) (Fig 3C). Such progressive changes would not have been well established within the time scale of the SOD1 pathology (which has onset at approximately 3 months with death occurring at 5 months). In future it would be useful to carry out the DAO^R199W^ cross with a lower copy number SOD1 mouse line.

Two recent studies have further supported the relevance of DAO dysfunction to ALS pathogenesis. Firstly in a comprehensive exome sequencing study in 2,874 cases with sporadic ALS and 6,405 controls, interestingly, it was shown that DAO was the only known predisposition gene where the presence of DNA variants was significantly associated with clinical outcome, decreasing rates of survival [29]. Secondly, in a study of the effect a pathogenic mutation in a gene encoding an RNA binding protein, hnRNP A2/B2, that, in common with mutations in the FALS gene VCP causes multisystem proteinopathy, it was found to have a profound effect on the splicing of DAO [30]. The resulting abnormality was a decrease in the constitutive form of DAO, the full length transcript analysed in this study (Fig 1), accompanied by an increase in the level of a transcript lacking exon 9, that leads to truncation of the protein and severely reduces enzyme activity. In future studies, it will be important to further elucidate whether the formation of this alternative transcript contributes to the pathogenesis of ALS.

## Materials and methods

All reagents purchased from Sigma unless indicated otherwise.

### Ethics statement

All animal experiments were carried out under license from the Home Office (UK) in accordance with the Animals Scientific Procedures Act 1986 and were approved by Imperial College London / Royal Veterinary College ethical review committees.

### Establishment of the DAO^R199W^ transgenic mouse line

Transgenic mouse lines overexpressing DAO^R199W^ were created using human DAO^R199W^, driven by a chicken β-actin promoter and cytomegalovirus enhancer, pCAGGS (Niwa et al., 1991). The linearized transgene was microinjected into pronuclei of fertilized eggs from C57BL6×CBA/Ca female mice, which were subsequently transferred to pseudopregnant recipients. Integration of the transgene into the genomic DNA was determined 3–4 weeks after birth, by collecting a small ear biopsy from the pups and screening for the transgene expression by using polymerase chain reaction (PCR). Hemizygote colonies from two independent founders expressing the transgenes were established and maintained by crossing DAOR199W males with wild-type F1 hybrid C57BL6xCBA/Ca females.

### Survival studies

Two transgenic mouse lines were used, a widely used high copy number SOD1^G93A^ and the DAO^R199W^ mouse lines, generated in this study. Mice overexpressing human SOD1 bearing the G93A mutation causing a motor neuron phenotype [B6SJL-TgN SOD1^G93A^ 1 Gur/J, Stock number 002726] [31] were initially purchased from the Jackson Laboratory (Bar Harbor, ME). They were maintained as hemizygotes by breeding male mice with wild type F1 hybrid C57BL6xCBA/Ca females. The SOD1^G93A^ mice have been generated on this background for over 28 generations, and animals on this background have similar disease characteristics (as previously assessed by hindlimb electrophysiological parameters [32, 33] and longitudinal monitoring of neurological score and body weight [34, 35] and life span to the original and more widely used B6SJL hybrid genetic background [36, 37]. We did not observe any changes in terms of lifespan and disease progression in these animals with successive generations in our laboratory [35, 38].

A double transgenic (DT) mouse model was generated following breeding of heterozygous high copy number SOD1^G93A^ males with heterozygous DAO^R199W^ females, generating 4 different genotypes per cross. Genotypes obtained from the cross were SOD1^G93A^, SOD1^G93A^/DAO^R199W^, DAO^R199W^, and Wild-type.

#### Genotyping

Genotyping was carried out following weaning, using an ear biopsy (~1.5-3mm^2^ in size). DNA extraction was performed by overnight incubation of the samples at 55°C in 1x homogenisation ear biopsy buffer (50**μ**l). After digestion, proteinase K (Invitrogen) was inactivated at 100°C and samples were screened for the expression of the transgenes of interest (SOD1^G93A^ or DAO^R199W^) using specific PCR conditions for each genotype as detailed in **S1 File.**

#### Design for survival studies

Based on the results of Scott et al [19] who defined the optimal conditions for carrying out survival studies involving the SOD1^G93A^ strain, we used 24 mice per experimental group per cohort (12 males and 12 females), same-gender litter matching, and excluded any animal who had experienced loss of *SOD1*^*G93A*^ transgene copy number [19]. Disease onset and progression were established through a combination of longitudinal monitoring of weight and neurological score [19, 34, 39]. Each individual animal was assessed using a simple neurological scoring system, which provided a rapid, non-invasive and unambiguous measure of disease onset and progression in SOD1^G93A^ mice [19, 34, 35, 38, 39]. Weight loss in high copy number SOD1^G93A^ mice is normally a gradual process that begins at approximately 90 days of age and continues until death. Rapid losses of weight over a short period of time that does not fit the normal pattern can therefore be indicative of non ALS related problems [19]. In addition, based on the data generated from the previous survival analyses in our laboratory we determined the end point as the inability of the animal to right itself within 20 seconds of being placed on its side. From 100 days of age, the righting reflex of each mouse was examined twice daily to ensure that the end point of each mouse was accurately determined. Once mice reached the end point, they were euthanized via cervical dislocation, the date and cause of their death were recorded and an ear and tail biopsy was collected for PCR and Q-PCR respectively to reconfirm the genotype and for examination of SOD1^G93A^ transgene copy number, as changes in the latter affect disease progression and ultimately survival. SOD1^G93A^ transgene copy number variation was assessed by quantitative PCR (qPCR) in any animal demonstrating outlying disease progression or survival characteristics [40].

Animals participating in all the *in vivo* studies were housed in a minimal disease facility at the Royal Veterinary College, with a ‘12:12’ hour ‘light:dark’ cycle, food and water *ad libitum*. Breeding trios each consisting of one SOD1^G93A^ transgenic male and two DAO^R199W^ females were set up simultaneously to generate a large cohort of age matched pups with a mixture of genotypes namely SOD1^G93A^, DAO^R199W^, SOD1^G93A^/DAO^R199W^, and wild type, for enrolment in the “SOD1^G93A^ x DAO^R199W^” survival study. Littermates of the same sex were housed together regardless of the genotype (not exceeding 5 animals per cage) to ensure consistency of the results in terms of age and gender, and the assessor was blinded to the genotypes of the individual mice until the termination of the study.

#### Survival study analysis

The development of ALS related disabilities were assessed by means of neurological scoring system for both hind legs twice weekly for each mouse from approximately 30 days of age in the case of “SOD1^G93A^ x DAO^R199W^” cross. Neurological scores were assigned using a scale of 0–4, established by the ALSTDI through detailed observations of SOD1^G93A^ mouse pathology [19, 34, 39]. Criteria used to assign each score level were:

- **0:** Full extension of hind legs away from lateral midline when mouse is suspended by its tail and mouse can hold this for 2 seconds, suspended 2–3 times.
- **1:** Partial or full collapse of leg extension towards lateral midline or trembling of hind legs during tail suspension.
- **2:** Toes curl under at least twice during walking of 12 inches or any part of foot is dragging along cage bottom (a clean cage containing no bedding was used for this analysis to permit easy viewing of the toes).
- **3:** Minimal joint movement or rigid paralysis, foot not being used for forward motion.
- **4:** Mouse cannot right itself within 20 seconds from one or both sides when placed on its side.

#### Graphical representation & statistical analysis

In order to demonstrate overall changes in body weight and neurological score, mean values for both body weight and neurological score were plotted for each group over time until the point in time at which the last mouse in the group reached the humane end point. To prevent decomposition of the mean weight and neurological score values as mice reached the end point, final weight and neurological score values for individual mice that reached the end point were carried forward until the time point at which the last mouse in that particular group reached the humane end point. This is a practice commonly used when performing survival analyses with longitudinal monitoring of weight and neurological score [34, 38, 39]. In order to avoid the loss of statistical validity that can occur when performing repeated analyses on correlated serial measurements for the same subjects, relevant summary measures for weight and neurological score data were selected as advised by Matthews and colleagues and statistical analyses were performed on these summary measures [21]. Summary measures selected include: time to attain peak weight (the earliest indicator of disease onset), time from peak weight to death (an indicator of disease progression), time to attain a neurological score of 2 in both hind-limbs (defined as the onset of neurological symptoms) and time to reach a score of 4 (the uniform humane end point selected for this study). These summary measures all represent ‘time to event measures’ and were analysed via Kaplan-Meier survival analyses: the log-rank test was performed to determine the statistical significance amongst experimental groups with the null-hypothesis that presence of the *DAO* transgene did not change the time taken to reach the event being analysed. A low P-value thus indicates that the differences between the curves did not occur due to chance, with the threshold for significance being set at (P < 0.05) for all analyses [38]. All Kaplan-Meier survival analyses were carried out using Graphpad Prism 6 software.

In the case of assessment of overall body weight, animals were weighed and values were plotted as ‘mean group body weight’ at each particular age using Excel software. Since each genotype group generated through the crosses was considered as a new strain of mice, and taking into account the fact that the differences in body weight commenced at birth, it was concluded by our statistician advisor that differences in body weight between the different experimental groups were not statistically comparable. Thus, no statistical test was carried out for this particular variable.

#### Gait analysis

Upon completion of the SOD1^G93A^/DAO^R199W^ survival study, animals expressing the *DAO*^*R199W*^ mutant transgene and their wild type littermates were recruited to a gait analysis study carried out using the DigiGait Imaging System (Mouse Specifics, Inc.). An automated system equipped with transparent treadmill belt and adjustable speed, and a video camera mounted underneath the belt captured the image of the ventral side of the animals automatically pixelating and vectorizing the ventral view of the animal [15]. The collected images were automatically analysed via the DigiGait software (Mouse Specifics, Inc.) where the area of each paw was recorded in correspondence to its movement and a periodic wave curve was therefore generated describing the advance and retreat of the four limbs in comparison to the treadmill belt through consecutive strides [15]. The portions of the paw that were in contact with the treadmill belt were automatically recognized as the stance phase of the stride by the software and the portion of the paw not in contact with the treadmill belt were consequently identified as the swing phase of the stride [15]. In addition, various other gait dynamics (i.e. over 40 parameters) were determined for each individual limb by the software that were extracted from the period of time spent in various portions of the walking phase, including paw area at peak stance, paw angle, brake, swing, stride length, and stepping frequency (**Text A in S1 File**).

#### Spinal cord collection & preparation

Animals were deeply anaesthetized by intraperitoneal (IP) administration of pentobarbital (Merial, UK) overdose (approximately 120–150μl). Under terminal anaesthesia the animals were subjected to transcardial perfusion with ice-cold PBS and ice-cold 4% PFA (prepared in PBS) respectively. Upon completion of the perfusion process, the spinal column was removed and post-fixed in 4% PFA at 4°C overnight. The spinal cord was then extracted from the spinal column and cryoprotected in 30% sterilized sucrose solution (prepared in PBS) at 4°C, a process that often required a few days during which the spinal cord sank towards the bottom the container within the sucrose solution. The lumbar region of the spinal cord was then selectively dissected, and embedded in gelatin and mounted on a cork block held in place with Cryo M-bed (Bright). This process involved vertically mounting the spinal cord on the cork block with the most caudal end placed on the cork block. The ventral side of the spinal cord was marked on the cork to allow orientation of the block within the cryostat such that the ventral side met the knife first [37]. The mounted spinal cord was then snap-frozen in liquid nitrogen cooled isopentane (Fisher Scientific) (-55°C, +/-5°C) and stored at -80°C.

#### Lumbar spinal cord sectioning

The lumbar regions of 23 mouse spinal cords were sectioned using a cryostat (Bright, UK). Sections of 20μm were taken between the regions of L2-L6. Sections were arranged on Superfrost miscroscope slides (VWR, Belgium). Slides with sections were frozen at -80°C pending analysis.

#### Staining of sections

Nissl staining of nucleic acids in the tissue was performed on every 6^th^ slide to identify the L3 to L5 regions of the animals’ spinal cords. Sections were placed in Nissl stain for 30 minutes before washing and dehydrating in graded alcohols before clearing in xylene and mounting with coverslips and DPX mountant (Sigma-Aldrich). The L3 to L5 region for each animal was determined using a Leica DM2500 microscope and descriptions of mouse spinal cord regions by Watson at al. [41]. The lumbar motor neuron pool starts at L3 and extends to L5 [42].

Immunohistochemistry for the enzyme ChAT was performed on every 2^nd^ and 5^th^ slide on the L3 through to L5 regions of animals. After rehydrating with phosphate buffered saline with 0.1% Tween®20 (Sigma-Aldrich) (PBS/T), endogenous peroxidase activity was quenched by incubating sections in PBS/T with 0.3% hydrogen peroxide (Sigma-Aldrich) for 5 minutes. Sections were then incubated with Avidin for 10 minutes and after washing with Biotin for 10 minutes, both from the Vector blocking kit (Vector, USA). Sections were washed again with PBS/T and blocked with 5% bovine serum albumin (Sigma-Aldrich) (BSA) in PBS/T for 30 minutes. Slides were washed with PBS/T and sections were incubated with the primary antibody, anti-ChAT (Millipore, USA), at 1:100 in PBS/T with 1% BSA overnight at 4°C.

After 3 x 5 minute washes with PBS/T sections were incubated in the secondary antibody, biotinylated anti-goat (Vector) at 1:500 in PBS/T with 1% BSA for 1 hour. Vector ABC reagent (Vector stain Elite kit) was made with 2 drops of solution A and 2 drops of solution B in 5ml PBS/T and left at room temperature for 30 minutes and then put on ice for 30 minutes before use. Sections were washed for 3 x 5 minutes with PBS/T and incubated in ABC reagent for 30 minutes. After 3 x 5 minute washes with PBS/T DAB solution was added made up using the DAB peroxidase substrate kit (Vector) of 2 drops of buffer, 4 drops DAB and 2 drops of hydrogen peroxide in 5 ml of distilled water. After 2 minutes DAB was washed off with water and dehydrated with graded alcohols before clearing in xylene for 2 x 10minutes. Slides were mounted with DPX and 60mm coverslips.

#### Motor neuron counts

For the ChAT stained slides the motor neurons of every section within the L3-L5 regions were counted. The motor neurons were counted that were identifiable in the structures of the lumbar ventral horn shown in Fig 3C [41]. Stained cells were included in the counts if they had a diameter of ≥20μm, specific labelling of the cytoplasm and a spherical nucleus with clear nucleolus. Cells were imaged with the Leica DM2500 microscope and the longest diameter of the cell body was measured using Q Imaging software. Counting was done blind to genotype of the animal and left and right sides of the spinal cord sections were counted separately. The 40 sections prior to the end of L5/start of L6 boundary were included in the total counts of motor neurons. Confirmatory counts were performed on every 12^th^ section within this region of the lumbar spinal cord using Nissl stained sections. When counting was completed, the sums for the left and right side of the spinal cord sections included for each animal were separated by genotype. Unpaired, one-tailed t-tests were performed with GraphPad Prism (USA) to identify significant differences between genotype groups, where p≤0.05.

#### Western blot analysis

All animal tissues were collected upon euthanization of the animal via cervical dislocation, snap-frozen in liquid nitrogen and stored at -80°C. Tissues were then weighed and ground in the presence of liquid nitrogen using a pestle and mortar, and incubated on ice for 30 minutes in chilled RIPA buffer solution (150 mM sodium chloride, 1.0% NP-40, 0.5% sodium deoxycholate, 0.1% SDS, 50 mM Tris, pH 8.0, containing 1mM PMSF, 1mM sodium orthovanadate, 5mM sodium fluoride, and a mixture of protease inhibitors (Roche). The samples were centrifuged at 4°C for 15 minutes at 15,000rpm, the supernatants were removed and used for protein quantification using the ‘BCA protein assay kit’ (Thermo Scientific) and the final concentration was determined using a ‘Nanodrop ND-1000 Spectrophotometer’ (Thermo Scientific) instrument. Samples were separated by 10% SDS/PAGE using NuPAGE LDS loading dye (Life Technologies), proteins transferred from the gel onto a PVDF membrane (Millipore) for 1 hour at a constant current of 80V, blocked in 5% non-fat milk/TBS-Tween (0.1%), and incubated with primary antibody for 1 hour at room temperature followed by secondary antibody for a further hour. All antibodies were prepared in TBS/T at the following dilutions: Anti-porcine DAO (Nordic, 1:2000); anti-beta tubulin (Abcam, 1:10000); goat anti-rabbit HRP (Bio Rad, 1:50000). Membranes were developed using enhanced chemiluminescence (‘Amersham Hyperfilm ECL’ (GE Healthcare)).

#### Quantitative PCR for DAO expression

Spinal cord, brain and quadriceps muscle samples were pulverised under liquid nitrogen and the powder mixed with TRIzol reagent at a ratio of 1ml TRIzol per 100mg tissue (quadriceps muscle) or 1ml per 50mg (brain and spinal cord). Samples were extracted from tissue samples using a TRIzol protocol as supplied by the manufacturers with the addition of an extra chloroform extraction step (1:1) after collection of the aqueous phase. RNA was assessed via nanodrop to ensure good 260/280 and 260/230 ratios (>1.9). Samples with 260/230 ratios lower than 1.9 were cleaned by a second round of isopropanol precipitation.

For cDNA synthesis, 800ng of RNA per reaction was reverse transcribed using the RTnanoscript2 kit (Primer Design) and final reactions were diluted 1/5 (empirically determined to be a sufficient dilution to prevent cDNA synthesis reagents having any effect on subsequent PCR efficiency). qPCR was performed in a CFX384 light cycler (Biorad) using PrecisionPLUS mastermix with SYBR green (Primer Design), with 10ul volumes of primers at a final concentration of 500nM, and with 10-15ng cDNA per well. Cycling conditions: 2mins at 95°C, then 50 cycles of: 15sec 95 ^o^C, 20sec 60°C, 20sec 72 ^o^C followed by a melt curve (60–95 ^o^C in 0.5 degree steps).

The following primers were used:

hDAO qPCR Ex8F: CACCCATGACCCAGAGAGAG
hDAO qPCR Ex9R: ATTCTTCAGTGTGGGCTCCA
mDAO qPCR Ex8F: CCCATGATCCTAGCCTTGGT
mDAO qPCR Ex9R: GCTCCAGTTTACAGCAGCTC

Pak1ip1 was used as a reference gene in all tissues (Primer Design).

### Determination of D-Amino Oxidase activity via spectrophotometric assay

DAO activity assays used a protocol modified from Tedeschi [14].

Non-transgenic (WT) mice and mice carrying the DAO^R199W^ transgene were killed by cervical dislocation and tissues harvested rapidly. 3–5 animals were used per genotype. Brains, spinal cords and quadriceps muscles were flash-frozen and then pulverised under liquid nitrogen using a mortar and pestle before storage at -80°C. Tissue homogenates were prepared by combining powdered tissues with extraction buffer (75mM Sodium Pyrophosphate pH 8.5, supplemented with 0.5% NP40, complete protease inhibitor cocktail (Roche) and 1% phosphatase inhibitor cocktails 2 and 3 (Roche)) to a final concentration of approx. 50mg.ml-1 and vortexing vigorously. Homogenates were incubated on ice for 10 minutes with occasional mixing before removal of insoluble material by centrifugation (13krpm, at 4°C in a benchtop microfuge). Soluble protein fractions were collected and protein concentrations (typically 5-10mg.ml-1) determined by DC protein assay (BioRad).

DAO spectrophotometric assays were performed in duplicate in flat-bottomed 96-well plates (Corning), using an M200 Pro plate reader (TECAN) at room temperature. Soluble protein fractions (typically 100-200ug of soluble protein per well) were added to assay buffer (75mM Sodium Pyrophosphate pH 8.5, 50mM D-serine, 20uM FAD, Horseradish Peroxidase (1U.ml-1), ~1mM o-dianisidine–shaken well to oxygenate), mixed briefly, and absorbance change at 436nM measure at approximately sixty second intervals for one hour. O-dianisidine exhibits poor solubility in most aqueous solutions. For these assays, a stock solution at 33mM was prepared in a 1:1 mix of DMSO and water, then added to 75mM sodium pyrophosphate assay buffer to a final concentration of 1mM. The solution was incubated at room temperature for 10 min to allow any insoluble dye to precipitate fully, then filtered before addition of FAD, D-Serine and peroxidase. Crude tissue isolates typically exhibit a lag phase of 2–10 minutes before reaching maximal rates, with rates subsequently lowering to a basal non-specific rate after 20–30 minutes. Maximum rates were recorded and compared against a standard curve of purified porcine DAO (loaded on the same plate).

## Supporting information

S1 File **Text A. Methodology: Genotyping and Gait Analysis. Figure A. DAO enzyme activity in spinal cord and brain from wild-type (WT) and DAO**^**R199W**^ **transgenic mice (R199W).** Activity measured as mUnits/mg tissue/lysate in brain and spinal cord from wild-type (WT) and DAO^R199W^ transgenic mice (R199W). **Figure B. D-serine immunoreactivity in lumbar spinal cord from control and DAO**^**R199W**^ **transgenic mice**. A rabbit polyclonal Anti-D-Serine antibody (Abcam) raised against d-serine cross-linked to BSA with glutaraldehyde was used at a dilution of 1 in 5,000 as previously described in detail [6]. **Figure C. Serine racemase immunoreactivity in lumbar spinal cord from control and DAO**^**R199W**^ **transgenic mice.** A mouse monoclonal Anti-serine racemase antibody (BD Biosciences) was used at a dilution of 1 in 1000 as previously described in detail [6].
(DOCX)
