# Supplementary material for: Characterisation of the pathogenic effects of the in vivo expression of an ALS-linked mutation in D-amino acid oxidase: Phenotype and loss of spinal cord motor neurons
Source: PLoS One. 2017 Dec 1;12(12):e0188912. doi: 10.1371/journal.pone.0188912 (PMC5711026; doi:10.1371/journal.pone.0188912)

**Supporting Information**

**S1 File:**

**Text A: Methodology: Genotyping and Gait Analysis**

**Genotyping**. SOD1^G93A^ PCR: thePCR reaction mixture for one single reaction consisted of: 9.35µl PCR-grade water, 2µl (10x) PCR buffer Mg free (Invitrogen), 2µl PCR Enhancer (Invitrogen), 0.8µl 2.5mM dNTPs (Invitrogen), 2µl (10 pmol/µl) Forward Primer (Invitrogen) , 2µl (10 pmol/µl) Reverse Primer (Invitrogen), 0.6µl (50mM) MgCl_2_ (Invitrogen),0.25µl Taq DNA Polymerase, recombinant (Invitrogen), 1µl DNA template (digested ear sample). Primer sequences for the amplification of SOD1^G93A^ transgene were: Forward Primer: 5’-CATCAGCCCTAATCCATCTGA-3’ and Reverse Primer: 5’-CGCGACTAACAATCAAAGTGA-3’. PCR Cycling Conditions were as follows, Initial denaturation at 95^o^C for 3 minutes, 35 cycles of 95°C for 30 seconds, 60°C for 30 seconds, 72°C for 45 seconds, 72°C for 2 minutes with a final hold temperature at 4°C.

DAO PCR: the PCR reaction mixture for one single reaction consisted of: 36.75µl PCR-grade water, 5µl (10x) PCR buffer (Qiagen), 2µl PCR Enhancer (Invitrogen), 4µl 2.5mM dNTPs (Invitrogen), 2µl (10 pmol/µl) Forward Primer (Invitrogen) , 2µl (10 pmol/µl) Reverse Primer (Invitrogen), 0.6µl (50mM) MgCl_2_ (Invitrogen),0.25µl Taq DNA Polymerase, recombinant (Invitrogen), 1µl DNA template (digested ear sample). Primer sequences for the SOD1^G93A^ transgene were: Forward Primer: 5’-CATCAGCCCTAATCCATCTGA-3’, Reverse Primer: 5’-CGCGACTAACAATCAAAGTGA-3’. PCR Cycling Conditions were as follows, Initial denaturation at 95^o^C for 3 minutes, 35 cycles of 95°C for 30 seconds, 60°C for 30 seconds, 72°C for 45 seconds, 72°C for 2 minutes with a final hold temperature at 4°C. Primer sequences for the amplification of DAO transgene, Forward Primer: 5’-CTGCTAACCATGTTCATGC-3’, Reverse Primer: 5’-GTGTTTGAAGGTCCAGTGC-3’. PCR Cycling Conditions were as follows, Initial denaturation at 95^o^C for 2 minutes, 35 cycles of 95°C for 30 seconds, 55°C for 30 seconds, 72°C for 45 seconds, final extension at 72°C for 3minutes with a final hold temperature at 4°C.

**Gait Analysis**

Prior to the study, a small pilot experiment was carried out using both male and female wild type littermates (*n*=3 per sex) who have previously participated in the “SOD1^G93A^ x DAO^R199W^” survival study. By carrying out this experiment we aimed to select a suitable and unified speed bearing in mind that gender, weight and age enforce an effect on overall gait performance of the animal, thus by randomly selecting these wild type littermates from our survival study we aimed to account for these factors across all the participating animal. To avoid any possible training and the possible consequent error in the interpretation of the final results, animals participating in the pilot study were not recruited in the final gait analysis. During the pilot investigation a range of various speeds were examined to determine an optimum speed under which animals of both sex can comfortably perform the walking task. The suitable treadmill speed was therefore determined to be 15cm/s, which was therefore employed for the gait study for both males and females. Each animal was monitored only once and for the maximum of 1 minute, where at least six complete strides were recorded.

Upon completion of the study the data generated by the DigiGait software was transferred onto an Excel file, values for each parameter for each matching limbs were averaged and animals carrying the *DAO^R199W^* transgene were compared to their wild type littermates for each parameter. The data set was initially subjected to normality test, following which should the data successfully pass the normality test a two-tailed, unpaired t-test statistical analysis was carried out on the data set, and if the data set did not pass the normality test, it was subjected to Mann-Whitney test, with the threshold for significance being set at (P < 0.05) for all analyses. All statistical analyses were performed using Graphpad Prism 6.

| Gait Index | Measurement Unit | Description |
| --- | --- | --- |
| Stride | Seconds | The sum of stance and swing duration (duration of one complete stride for one paw). |
| Stance | Seconds | The weight bearing portion of the stride in which the paw remains in contact with the belt. |
| Swing | Seconds | The portion of the stride in which the paw is not in contact with the belt. |
| Brake | Seconds | The time interval between initial and maximal paw contacts with the belt. |
| Paw Angle | Degree | Angle of the paw during peak stance in relation to the long axis of the body. |
| Paw Area at Peak Stance | Cm^2^ | The maximal paw area in contact with the treadmill during the stance phase of the step cycle. |

**Table 1.** Definition of DigiGait parameters for a single limb.

Descriptions of gait parameters used to evaluate changes in gait for the DAO^R199W^ mutant males and females versus their wild type littermates.

**Figure A. DAO enzyme activity mUnits/mg tissue/lysate in brain and spinal cord from wild-type (WT) and DAO^R199W^ transgenic mice (R199W)**


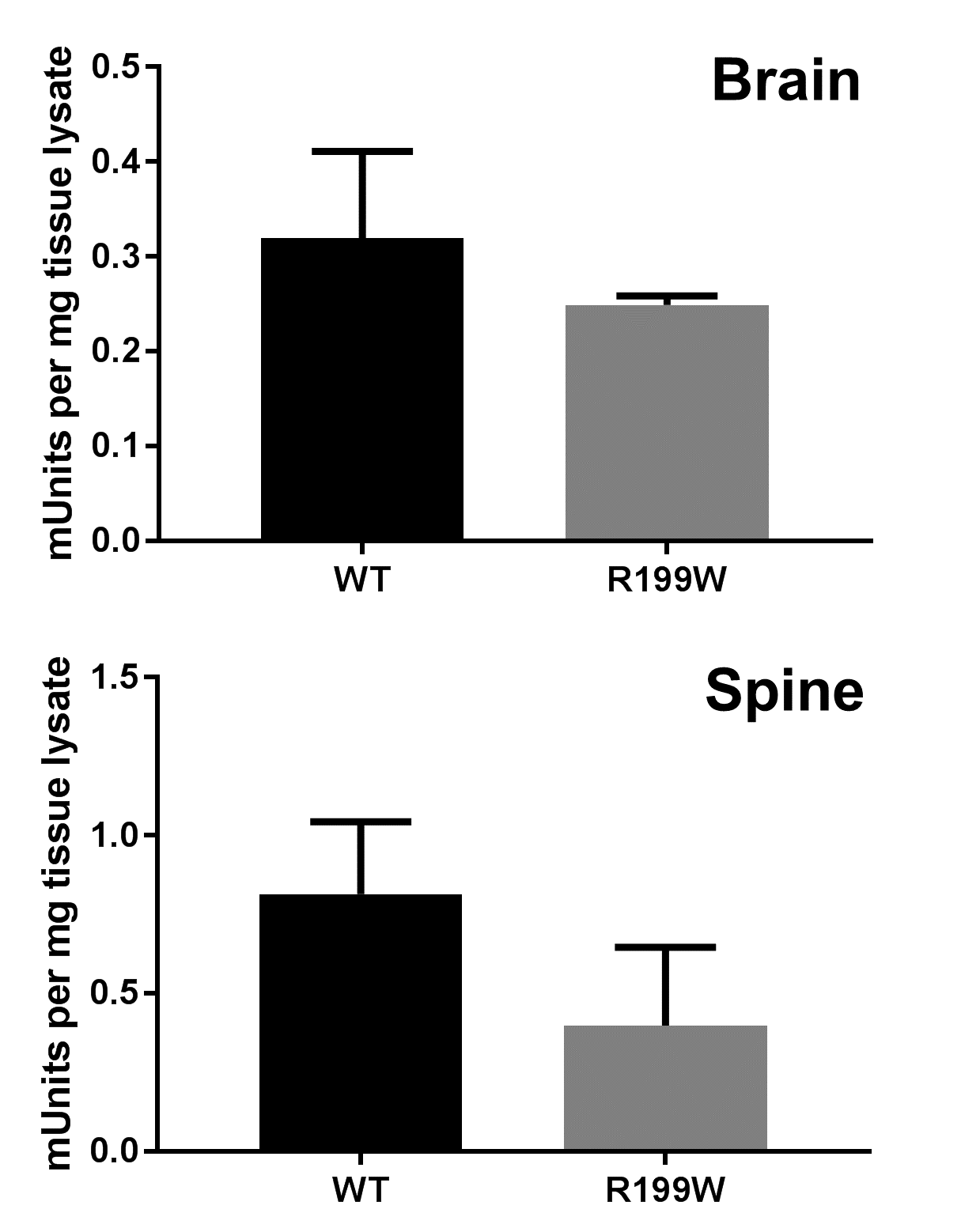


**Figure B.**  **D-serine immunoreactivity in lumbar spinal cord from control and DAOR199W transgenic mice.**

A rabbit polyclonal Anti-D-Serine antibody (Abcam) raised against d-serine cross-linked to BSA with glutaraldehyde was used at a dilution of 1 in 5,000 as previously described in detail [6].


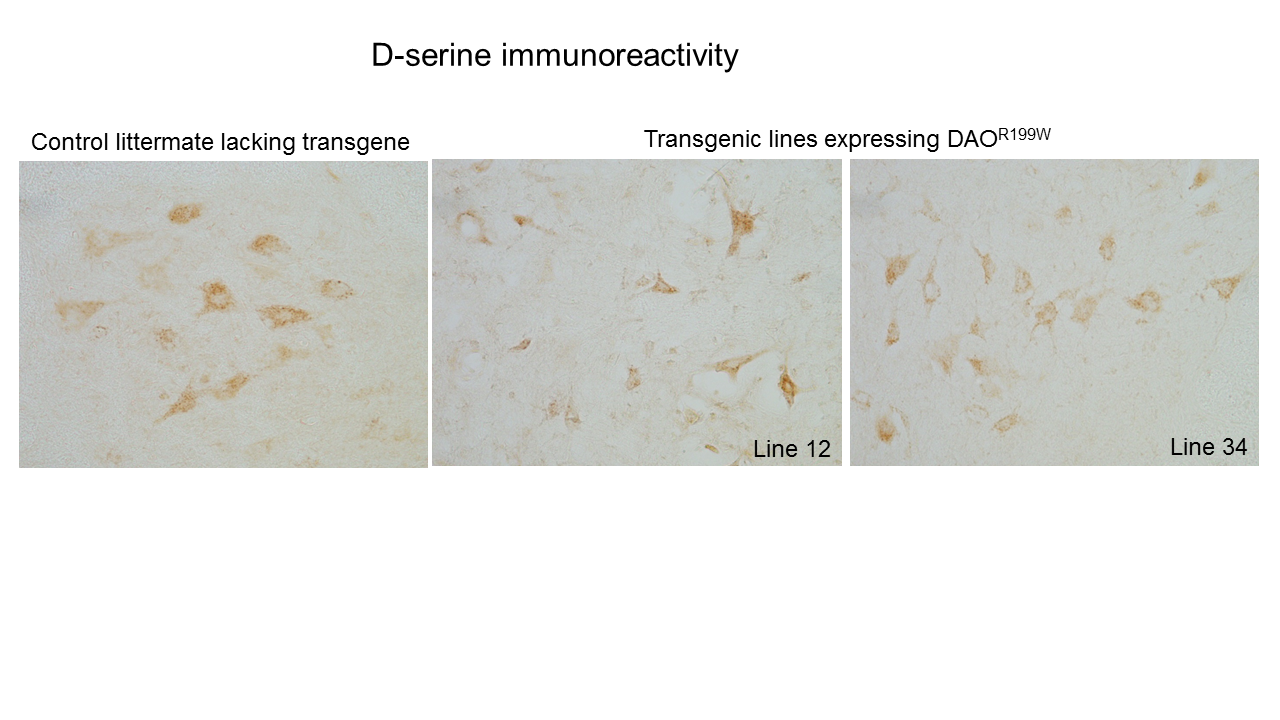


**Figure C. Serine racemase immunoreactivity in lumbar spinal cord from control (2 magnifications) and two DAOR199W transgenic mouse lines.**

A mouse monoclonal Anti-serine racemase antibody (BD Biosciences) was used at a dilution of 1 in 1000 as previously described in detail [6].


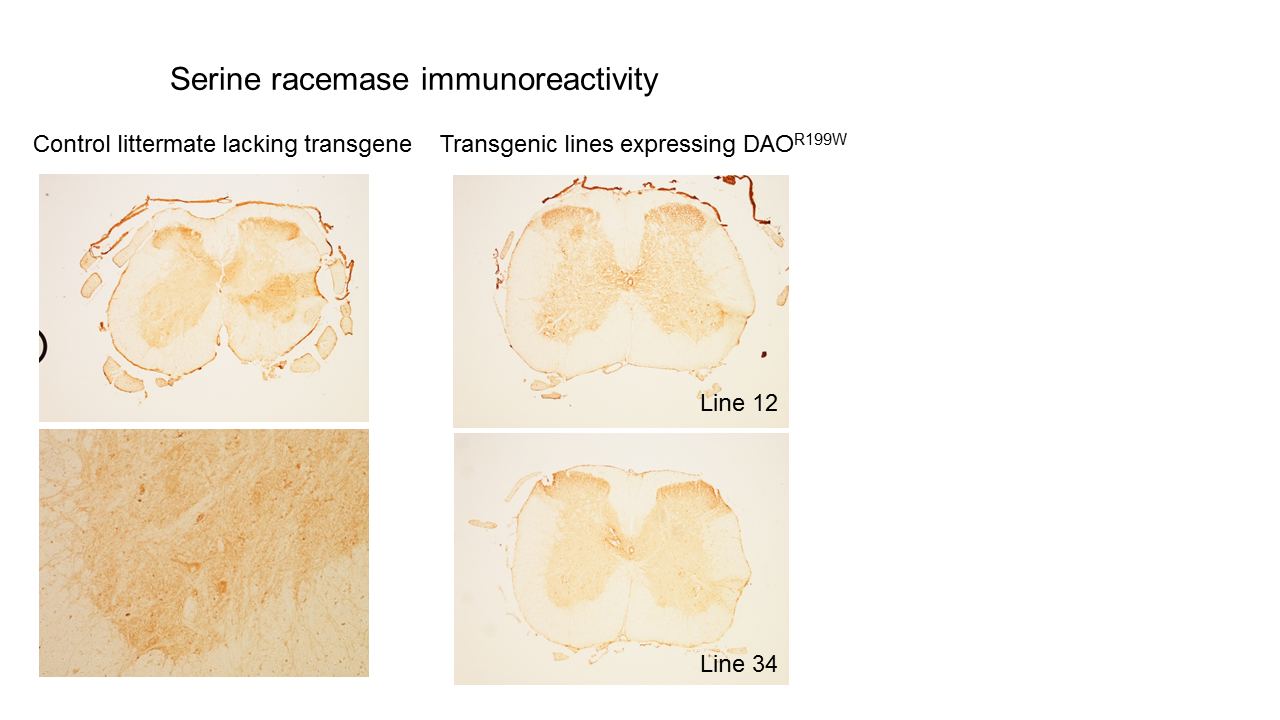

Supplement: S1 File — Text A. Methodology: Genotyping and Gait Analysis. Figure A. DAO enzyme activity in spinal cord and brain from wild-type (WT) and DAOR199W transgenic mice (R199W). Activity measured as mUnits/mg tissue/lysate in brain and spinal cord from wild-type (WT) and DAOR199W transgenic mice (R199W). Figure B. D-serine immunoreactivity in lumbar spinal cord from control and DAOR199W transgenic mice. A rabbit polyclonal Anti-D-Serine antibody (Abcam) raised against d-serine cross-linked to BSA with glutaraldehyde was used at a dilution of 1 in 5,000 as previously described in detail [6]. Figure C. Serine racemase immunoreactivity in lumbar spinal cord from control and DAOR199W transgenic mice. A mouse monoclonal Anti-serine racemase antibody (BD Biosciences) was used at a dilution of 1 in 1000 as previously described in detail [6]. (DOCX) [file pone.0188912.s001.docx]
